# Effects of a sugar-sweetened beverage tax on prices and affordability of soft drinks in Chile: A time series analysis

**DOI:** 10.1016/j.socscimed.2019.112708

**Published:** 2020-01

**Authors:** Cristóbal Cuadrado, Jocelyn Dunstan, Nicolas Silva-Illanes, Andrew J. Mirelman, Ryota Nakamura, Marc Suhrcke

**Affiliations:** a Escuela de Salud Pública, Universidad de Chile, Chile; b Centre for Health Economics, University of York, UK; c Hitotsubashi Institute for Advanced Study, Hitotsubashi University, Japan; d Luxembourg Institute of Socio-economic Research, Luxembourg

**Keywords:** Chile, Sugar-sweetened beverage, Soda, Soft drink, Tax, Fiscal policy, Taxation, Price, Affordability

## Abstract

Chile is one of several countries that recently implemented a fiscal policy to reduce soft drink (SD) intake and obesity. In 2014 the government increased the existing ad-valorem tax on high-sugar SD by 5% and decreased by 3% the tax on low-sugar SD, based on a 6.25gr/100 ml sugar threshold. This study aims to evaluate the tax modification passed-on to consumers through prices, and to calculate changes in affordability of SDs. We analysed nationally representative consumer price index data of 41 soft drinks within 6 beverage categories between 2009 and 2016. Price change post-tax implementation was estimated for different categories (carbonates, juices, concentrates, waters and energy-sport drinks), using time-series analyses. In addition, changes in affordability were evaluated by estimating the changes in prices relative to wages. The price of carbonates increased by 5.60% (CI 95% 3.18–8.03%) immediately after the tax was implemented. A sustained increase in the prices of concentrates was observed after the implementation. Unexpectedly, a smaller increase was also seen for the price of bottled water – a category that saw no tax change. There were no effects for juices and energy-sports drinks. There was a reduction in affordability for carbonates, concentrates and waters. Overall, the fiscal policy was effective in increasing prices and there are some signs of reduced affordability. Results varied substantially among categories directly affected by the tax policy. While for carbonates the price increase exceeded the tax change (‘over-shifting’), in other categories subject to a tax cut, a price reduction was expected but the opposite occurred. As the effect of the tax on prices differed between categories, the effects of the tax policy on consumption patterns are likely to be mixed. Our findings underline the need to better understand and anticipate price setting behaviour of firms in response to a tax.

## Highlights

• Chile implemented a tax policy on soft drinks (SD) to promote healthier diets in 2014.
• It consists in a 5% tax increase on high-sugar SD and 3% reduction on low-sugar SD.
• Using consumer price index data we estimate policy impact on SD price/affordability.
• Overall, the policy was effective to increase prices and reduce affordability.
• Results varied substantially among SD categories, with some counterintuitive effects.

## 1 Introduction

The consumption of sugar-sweetened beverages (SSB) has been identified as one of the leading proximate drivers of the obesity epidemic (Basu et al., 2013), and Chile is one of the largest consumers of SSBs in the world. According to data from the Euromonitor Passport Database, Chile ranked first among a global sample of countries in terms of SSB calories sold per capita per day in 2014 (Popkin and Hawkes, 2016). Moreover, evidence shows that SSB consumption in Chile has been rapidly increasing, with the fastest growth in the world between 2009 and 2014 (Popkin and Hawkes, 2016). High levels of SSB intake were also observed in data collected by the National Survey on Food Consumption between 2010 and 2012, particularly among school-aged children (Araneda et al., 2015).

There is already a considerable literature studying the relationship between SSB consumption and weight gain. In one comprehensive study, Malik et al. (Malik et al., 2013) synthesized 22 cohort studies and 10 randomized control trials, finding systematic positive associations between SSB consumption and body weight in adults and children. Another meta-analysis found that people do not compensate the caloric contribution of SSB by reducing the intake of other food groups (Vartanian et al., 2007). Body weight gain is associated with increased risk of diabetes mellitus (Bell et al., 2014; Cloostermans et al., 2015; Guh et al., 2009), cardiovascular diseases (Mongraw-Chaffin et al., 2015; Ni Mhurchu et al., 2004; Owen et al., 2015; Rogers et al., 2007; Strazzullo et al., 2010; Wormser et al., 2011), chronic kidney diseases (Tanner et al., 2012; Thomas et al., 2011), and several types of cancer (Arnold et al., 2014; Renehan et al., 2010; Renehan et al., 2008). Moreover, SSB consumption has been linked to the incidence of type II diabetes and hypertension, even after adjusting for weight gain (Imamura et al., 2015; Jayalath et al., 2015).

There is growing interest in the importance of prices for controlling soft drinks consumption. A standard metric to assess changes in purchasing as a response to changes in prices is the price elasticity of the demand, and several studies show that SSB consumption is responsive to price changes, ranging from inelastic (<1) to moderately elastic estimates (>1) (Andreyeva et al., 2010; Cornelsen et al., 2015; Green et al., 2013; Powell et al., 2013). More elastic responses are generally observed in low socioeconomic groups and more competitive markets. In the Chilean market, the price elasticity of carbonates and ready-to-drink beverages was estimated to be even higher at −1.37 (Guerrero-López et al., 2017) and −1.3 (Caro et al., 2017) respectively, with the purchasing among the lowest income quintile being particularly sensitive to price changes (Caro et al., 2017; Guerrero-López et al., 2017).

Purchasing patterns are not only influenced by prices, but also by other factors, including consumer income. For this reason, a consideration of affordability – a measure that combines both prices and income in a single indicator – has been proposed as a relevant approach (E. Blecher, Liber, Drope, Nguyen and Stoklosa, 2017; Colchero et al., 2019; Paraje and Pincheira, 2018). Affordability can be defined as the amount of a certain good that is purchasable based on available consumer resources. For SSB products, it has been increasing globally in the last decades (E. Blecher et al., 2017; Colchero et al., 2019; Paraje and Pincheira, 2018), and particularly in Latin America (Colchero et al., 2019; Paraje and Pincheira, 2018). It is not surprising that in the same period, globally increasing trends in SSB consumption have also been observed (Popkin and Hawkes, 2016), with recent evidence suggesting an association between SSB affordability and obesity prevalence across countries (Ferreti and Mariani, 2019).

A growing number of countries has implemented tax policies in order to reduce soft drink consumption. Evaluations of these policies have been published for Mexico (Batis et al., 2016; Colchero et al., 2016), the United States (the city of Berkeley and Philadelphia) (Cawley et al., 2019; Falbe et al., 2015; Silver et al., 2017; Zhong et al., 2018), Barbados (Alvarado et al., 2019), Denmark (Smed et al., 2016), Hungary (Bíró, 2015), France (Berardi et al., 2016; Etilé et al., 2018) and Spain (Catalonia) (Castelló and Lopez Casasnovas, 2018). Although the implementation of these policies differs by type of tax (excise or ad valorem), taxed products, or in the way the tax was collected; previous research (Batis et al., 2016; Berardi et al., 2016; Bíró, 2015; Colchero et al., 2016; Etilé et al., 2018; Falbe et al., 2015; Silver et al., 2017; Smed et al., 2016) has mostly found these policies to be effective in increasing consumer prices, as intended in order to disincentivise consumption. Nevertheless, none of the previous research appears to have evaluated the impact of the policy on affordability.

In March 2014, Chile announced a tax reform to the *Impuesto Adicional a las Bebidas Analcohólicas* (IABA, or “additional tax on soft drinks”), an ad valorem tax implemented since 1933 with the last important modification coming in 1976. The tax reform was enacted in October 2014, affecting any non-alcoholic beverages to which colourants, flavourings or sweeteners have been added. For beverages above a sugar concentration of at least 6.25 gr per 100 ml (or equivalent proportion), the tax was increased from 13% to 18%, while for those below this threshold the tax was decreased to 10%, producing an 8% point tax difference between these beverage groups.(Biblioteca del Congreso Nacional de Chile, 2014). This is a particular characteristic from the Chilean ad valorem tax, where a mixed tax modification including a tax increase and a tax reduction based on a sugar threshold was implemented simultaneously. Non-alcoholic beverages without added sugar, such as plain water and dairy products, are not affected by the ad valorem tax. Energy and sports drinks were not affected by IABA prior to October 2014. Therefore – as part of IABA – they were subject to 10% or 18% tax increase depending on their added sugar content. The intervention was implemented nationwide at the same time and was part of a large tax reform including changes in other taxes such as those on tobacco, alcohol, green taxes and corporate income taxes.

Two studies have most recently evaluated the impact of this tax reform on SSB prices in urban areas using data from take-home purchases (Caro et al., 2018; Nakamura et al., 2018). Overall, both analyses found qualitatively similar results, but differed in the magnitude of the point estimate. Both studies found an increase in the prices of the products that were affected by a tax increase and a drop in the price of beverages subject to a tax reduction. The analyses of price using purchasing data suffer from a number of potential biases. First, since they rely on transaction records of consumers, the data cover only the price at which purchases were made (i.e. only prices that were reasonable and affordable to consumers). Such price data may understate the consumer price. Second, the previous studies focus on prices based on purchases made in urban areas only. Finally, the data could suffer from measurement error because consumers may not always report purchases accurately.

In this study, we evaluated the trends in the market prices before and after the implementation of the tax reform in Chile. Compared to the aforementioned studies, our analysis used price data that were posted in store, rather than reported or recorded by respondents. The data were nationally representative and collected by the Chilean National Institute of Statistics to estimate the consumers price index and inflation on a compulsory basis, hence there would be little measurement errors if any. In addition, we analyse the population level effects of the tax change on a measure of affordability of different non-alcoholic beverages, by comparing changes in prices relative to changes in wages before and after the SSB tax implementation.

## 2 Methods

We employ a quasi-experimental design to evaluate the effect of the SSB tax policy in Chile. We analysed the following six soft drink categories: carbonated drinks (sodas), juices, concentrates (powder juices), bottled waters, energy and sports drinks, and milk. Carbonated drinks, juices, concentrates, and energy drinks were affected by the tax policy. While a small proportion of bottled waters (i.e. flavoured waters) were affected by the tax modification, most of the products in this category (i.e. plain water) were unaffected. Milk and dairy products were excluded from the taxation, regardless of their added sugar content. Furthermore, other non-beverage products that were unaffected by the policy were analysed separately for comparison purposes. These included: dish detergents, laundry detergents, napkins and toilet paper.

### 2.1 Data sources and variables

Aggregated longitudinal monthly data of prices for each product category were obtained from the Laspeyres Consumer Price Index (CPI), provided by the Chilean National Institute of Statistics for the 93-month period between January 2009 and September 2016. We limited the data used for the analysis to two years after the policy implementation (24 months) to reduce a potential bias in the estimated effects that might result from other policies implemented after the tax (such as the front of package labelling law). Price data were collected every two weeks in 15 major cities (regional capitals), incorporating 9629 stores in the sample. Products were sampled in proportion to their frequency of consumption and their expenditure share within the household budget based on nationally representative surveys. From this micro-level price data, an aggregate Laspeyres Price Index is calculated for each food and beverage product category by estimating an arithmetic mean weighted by the expenditure share of each product (e.g. soda of a particular brand) over the overall beverage consumption category (e.g. carbonates) observed in a nationally representative sample of households (Instituto Nacional de Estadísticas, 2013). Price data were continuously monitored using this method, ensuring comparability of the estimates across periods. A total of 41 soft drinks were arranged into the 6 previously listed categories. Since the database does not contain information on the sugar content of each soft drink, it was not possible to discriminate the tax rate for each product category. From other sources (see Supporting Information – SI1 – Market share and expected price increase), we know that 87.9% of the carbonates and 85.6% of the juices were affected by the tax increase (from a tax rate of 13%–18%), while 98.1% of the concentrates were affected by a tax reduction (from a tax rate of 13%–10%). In the case of bottled waters, 58.6% of the volume purchased were plain waters (no tax modification) and 41% within the flavoured low-calorie group (reduction from a tax rate of 13%–10%). Since the CPI data were proportionally sampled based on the market share of each product within a category, we expect that the average price change for each category should follow a similar distribution.

In order to estimate the change in affordability of soft drinks, prices for these products were compared with general trends in the purchasing power of the population. Data on wages were used (again from the National Institute of Statistics) comprising monthly survey data of wages from all economic sectors in Chile (Instituto Nacional de Estadísticas (INE), 2017). Finally, data on national unemployment rates from the National Survey of Employment (Instituto Nacional de Estadísticas, 2017) were obtained in order to account for external macroeconomic factors that could affect both prices and affordability.

#### 2.1.1 Soft drink market prices

The outcome variable of our main analysis is the price index (p_it_) for each product *i* in month *t*. The prices represent the market prices (as faced by consumers) of the most frequently purchased products within the soft drinks in supermarkets and other grocery stores. Importantly, the prices capture potential discounts offered by the stores. In Chile, the Value Added Tax (VAT) is already reflected in the posted prices. To account for real price changes within the observed period, nominal prices were adjusted by the national inflation rate. The January 2014 price data was used as the base period for the CPI estimates.

Pass-through was defined as the magnitude of the tax change that was effectively transferred to the CPI. Based on the observed changes in prices, we estimated the pass-through resulting from the tax for each category. The effect of the tax was described as over-shifting when the ratio between the difference in prices and the difference in tax rates was greater than 1; conversely, under-shifting was defined as a ratio of less than 1.

To have a reference value of pass-through (i.e. a theoretical price level assuming a 100% pass-through) in each product category, we estimated a weighted average of the expected price change based on the share of products by tax rate in each category assuming a complete pass-through of the tax (See Supporting Information – SI1 – Market share and expected price increase). No data for the distribution of the sports and energy drinks data was available. Nevertheless, this category had a 0% rate before the tax, therefore all sports and energy drinks were subject to either a 10% or 18% net increase in the tax rate, and we assumed 10% as the most conservative estimate. Based on these calculations, we expected the following net price effects per CPI category: carbonates 4.0%; concentrates −2.8%; juices 3.8%; bottled waters −1.2%; sports and energy drinks 18%.

#### 2.1.2 Soft drink affordability

Following approaches used in the related literature (Bandi et al., 2013; Hancock, 1993; Rabinovich et al., 2009; Seabrook, 2010), we defined affordability as the amount of a certain good that is purchasable based on available consumer resources. Therefore, the affordability of a good is a function of the price of the good, the prices of other competing goods (for example other food and beverage categories) and the consumer disposable income. Adjusting the nominal price of the good by the increase in prices of other products based on the general inflation rate accounts for relative price changes over time. Nevertheless, the use of real prices does not incorporate the effect of income; therefore, increases in real prices do not necessarily imply that beverages become less affordable. To account for this, we estimated the effect of the tax on the affordability of beverages by adjusting the real price of products by the average wages in each time period. Previous international studies, mainly addressing tobacco affordability measures, used either GDP per capita (E. H. Blecher, van Walbeek and van Walbeek, 2004; E. Blecher et al., 2017) or weighed averages of wages (Guindon et al., 2002) as a proxy of purchasing power. To estimate the trends in affordability, we used the weighted average of wages based on a monthly national representative sample of different sectors of the Chilean economy (Instituto Nacional de Estadísticas, 2017). The choice to use this source was based on data sensitivity to short term variations, hence being more likely to capture the dynamic variability in the purchasing power trends. The affordability index ($a_{\text{it}})$ that we used is the ratio between wages and the price index (w_t_/p_it_), where w_t_ is the national wage index for each month *t* and p_it_ is the real price index for the product category *i* for each month *t*. Upward (downward) secular trends in this index can be interpreted as an increase (decrease) in the affordability of the product *i*. This measure is analogous to the affordability index used in a previous study on alcoholic beverages (Rabinovich et al., 2009).

### 2.2 Analysis

Due to the dynamic nature of the data and the outcome variables, the usual assumptions of independence needed for standard regression models are unlikely to hold. For example, price trends tend to show a high degree of autocorrelation, i.e. prices in previous months affect the price today. Dynamic regression models, that allow the inclusion of lagged variables to incorporate feedback over time, are preferred to obtain unbiased estimates. We used autoregressive Integrated Moving Average (ARIMA) models with exogenous variables (ARIMAX), incorporating dummy variables assigning pre/post-intervention periods and continuous variables to estimate differences in pre/post-intervention trends. Univariate series were analysed for each product in order to test the taxation effect. For these models, the parameter identification followed the Box-Jenkins approach (Box et al., 2008) to identify suitable models. Diagnostic checks confirmed white noise of the residuals (See Supporting Information – SI2 – Model diagnostics). In case of multiple feasible competing models, goodness of fit and parsimonious criteria was used to select the best model (Akaike information criterion and Bayesian information criterion). Wages and unemployment rates were incorporated in the regression model as continuous variables capturing macroeconomic conditions.

ARIMAX (p,d,q) models for price ($p_{\text{it}})$ and affordability ($a_{\text{it}})$ are defined as:

$${\text{Δ}}^{d}p_{\text{it}}=\beta_{i}\;Tax+\phi \;Time_{t}+\lambda \;\left(Tax*Time_{t}\right)+\;X_{t}+\mu_{i}+{\text{ n}}_{it\;}\text{ ,}$$

where ${\text{Δ}}^{d}p_{\text{it}}$ is the time difference of order d of the real price index (or affordability index) per product category *i* in month *t*. *Tax* is the policy dummy variable taking the value of 1 in the period starting in October 2014 and 0 before, and the parameter $\beta_{i}$ captures the average effect of the tax on price or affordability for each product. The parameter ϕ is the general time trend (continuous monthly variable starting in January 2009 until the data period). The parameter λ indicate changes in the trend of the time-series after the implementation of the tax, suggesting potential further medium-term effects of the policy. $X_{t}$ is the polynomial of economic adjustment variables wages and unemployment rates for each month. The term ${\text{n}}_{it\;}$ represents a polynomial of autoregressive (AR) and moving average (MA) parameters of the fitted ARIMA model:

$${\text{n}}_{it\;}=\overset{p}{\underset{j}{\sum }}\varphi_{i}y_{it-j}+\overset{q}{\underset{l}{\sum }}\theta_{l}\varepsilon_{it-l}+\;\varepsilon_{it}.,$$

where $\varepsilon_{it}$ is a white noise process, $\varphi_{i}$ is the AR parameter of order p (p is the number of lags in the dependent variable) and $\theta_{l}$ is the MA parameter of order q (q is the number of lagged error terms).

To test for a potential anticipatory response by the industry, we estimated announcement effects incorporating a dummy variable taking on the value of 1 in the period starting in April 2014 and 0 before, using ARIMAX models. As a sensitivity test, we explored potential breaks in the data using the approach proposed by Chen and Liu (Chen and Liu, 1993), to empirically account for unexpected changes in univariate time-series searching for level effects and temporary changes in each product time-series at different times than the implementation of the policy (see Supporting information – SI3 – Placebo test). Furthermore, ARIMAX model were fitted for other products unaffected by the policy (milks, dish detergents, laundry detergents, napkins and toilet paper) to discard potential unexpected effects (see Supporting information – SI5 - Sensitivity analysis).

Additionally, to check the robustness of our ARIMAX models results, fixed-effect and mixed-effect panel data regression models using different estimation methods were implemented to explore the sensitivity of the results to different estimation approaches.). Finally, models were also adjusted by food inflation were also included to test the robustness of our estimates (see Supporting information – SI5 - Sensitivity analysis).

Statistical analysis was performed using STATA 14.1 (Stata Corp, College Station, TX) and R 3.3.2. Datasets, R scripts and do-files are available upon request from the authors.

## 3 Results

### 3.1 Effects on prices

**Fig. 1:**
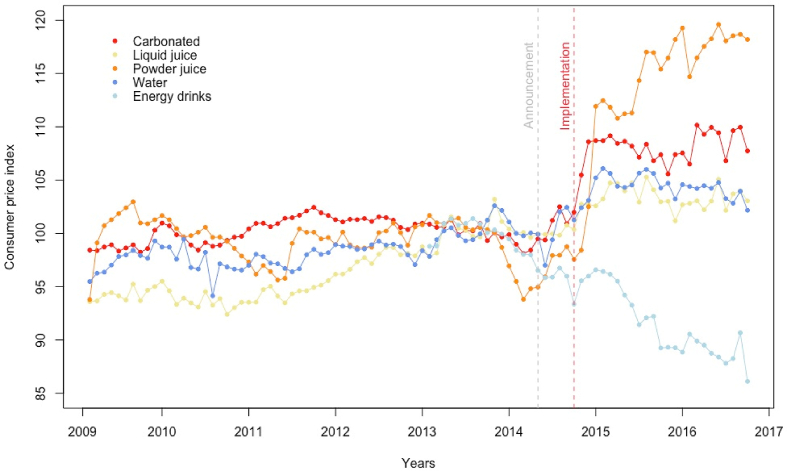
Trends in soft drinks prices 2009–2016, CHILE*. * Carbonates, juices, sports and energy drinks were affected by a tax increase (expected price effect of 4.0%, 3.8% and 18%, respectively), while bottled waters and concentrates were affected by a tax decrease (expected price effect of −1.2% and −2.8%, respectively). Grey line: policy announcement. Red line: policy implementation. (For interpretation of the references to colour in this figure legend, the reader is referred to the Web version of this article.)

Fig. 1 shows the descriptive trend of soft drink prices for the period 2009–2016. For all product categories, there were stable price trends in the pre-tax period. We found visible changes for some categories after the announcement and after the implementation of the tax policy. These effects are most noticeable for carbonates and concentrates. For energy drinks, we observed a stable downward trend for the entire period (note that price data for this category was available only from January 2013 onwards).

In our regression analysis, we found a consistent price level increase immediately after the tax implementation only for the carbonates and bottled water products (Table 1). In our fully specified ARIMAX model, carbonate prices increased by 5.60% (CI 95% 3.18–8.05%) after the tax implementation, without further changes in the general trend of prices observed in the pre- and post-tax period. For liquid juices, no consistent effects were observed, with a non-significant increase immediately after the implementation in the complete model including adjustment for potential announcement effects. For this category, the long-term trend changed after the tax, suggesting a gradual reduction in prices after the policy came into effect. In the case of concentrates, no effect was observed for the tax implementation, but an upward trend change was evident in the post-tax period. Further analyses corroborated that the price increase was evident only with a 2–3 month lag post-implementation (See Supporting Information – SI5 – Sensitivity analysis). We did not detect a significant announcement effect of the policy, with the exception of liquid juices where a price reduction was observed. Interestingly, for bottled waters, a category that was theoretically not affected by the tax increase due to it sugar content, we found a small yet significant price increase of 2.55% (CI 95% 0.74–4.35%) upon tax implementation, but with a negative price trend in the post-implementation period that attenuates over time. Energy and sports drink prices were unaffected by the policy immediately after implementation, even with a large tax increase on this category (up to 18% points) was introduced. Nevertheless, an attenuation of the long-term trend on price reduction on these products was observed in the post-tax period.

**Table 1:** Price effect (% change) on SOFT drink after the taxation implemented in Chile.

	Carbonates		Juices		Concentrates		Bottled water		Energy and sport drinks	
	Model 1	Model 2	Model 1	Model 2	Model 1	Model 2	Model 1	Model 2	Model 1	Model 2
Implementation effect	4.55*	5.60*	2.12*	0.52	−0.01	−0.17	2.89*	2.55*	2.16*	0.29
	[2.77; 6.33]	[3.18; 8.03]	[0.55; 3.69]	[-1.19; 2.24]	[-2.93; 2.91]	[-4.28; 3.95]	[1.25; 4.53]	[0.74; 4.35]	[0.08; 4.23]	[-2.78; 3.35]
Announcement effect		1.02		−1.98*		−0.16		−0.68		−1.72
		[-0.62; 2.67]		[-3.63; −0.33]		[-3.09; 2.78]		[-2.25; 0.90]		[-3.79; 0.35]
Time trend (monthly)	−0.04	−0.06	0.11	0.11	0.02	0.02	0.04	0.04	−0.54*	−0.43*
	[-0.29; 0.20]	[-0.30; 0.20]	[-0.08; 0.31]	[-0.08; 0.31]	[-0.53; 0.56]	[-0.53; 0.57]	[-0.17; 0.26]	[-0.18; 0.25]	[-0.95; −0.13]	[-0.84; −0.01]
Post-tax trend (monthly)	0.13	0.14	−0.20*	−0.25*	0.94*	0.94*	−0.13*	−0.14*	−0.04	−0.14
	[-0.20; 0.46]	[-0.18; 0.47]	[-0.35; −0.06]	[-0.35; −0.15]	[0.07; 1.81]	[0.06; 1.81]	[-0.25; −0.02]	[-0.25; −0.03]	[-0.42; 0.34]	[-0.49; 0.23]
Selected ARIMA model	(1,1,0)	(1,1,0)	(1,0,1)	(1,0,0)	(0,1,1)	(0,1,1)	(1,0,0)	(1,0,0)	(1,0,0)	(1,0,0)

Model 1: ARIMAX model without announcement effect. Model 2: ARIMAX model with announcement effect. Point estimates in percent change. 95% Confidence Intervals in parenthesis. *p < 0.05. ^♦^ Expected change in prices per category for pass-through estimates were: carbonates 4.0%; concentrates −2.8%; juices 3.8%; bottled waters −1.2%; sports and energy drinks 18%.

As expected, and providing more confidence in our findings, the analysis for milk and other non-taxed unrelated categories such as dish detergents, laundry detergents, napkins and toilet paper showed no consistent price-effects after the tax announcement or implementation (See Supporting information – SI5 Sensitivity analysis).

### 3.2 Effects on affordability

**Fig. 2:**
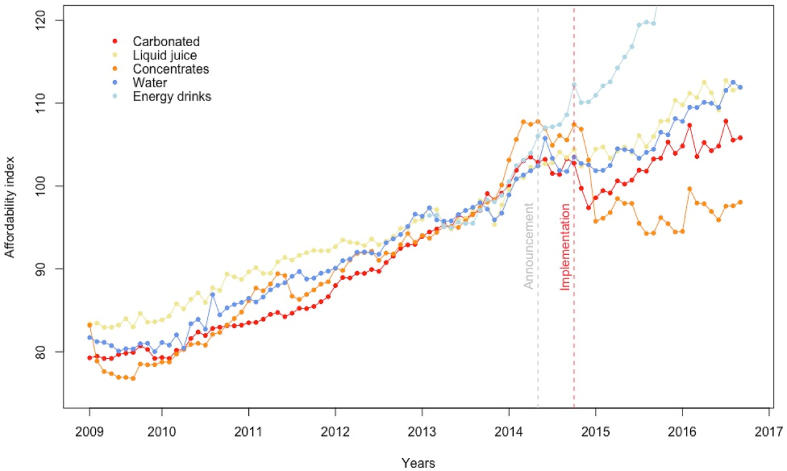
Trends in soft drinks affordability 2009–2016, Chile. Grey line: policy announcement. Red line: policy implementation. . (For interpretation of the references to colour in this figure legend, the reader is referred to the Web version of this article.)

Regarding affordability of beverage products, the descriptive Fig. 2 shows a general upward trend for all product categories in the pre-tax period. Since relatively stable trends in real prices were observed in this period (Fig. 1), the increasing affordability should be attributable mainly to wage increases. After an apparent decrease in affordability on the carbonates and concentrates groups, similar post-tax implementation upward (and parallel) trends as in the pre-tax period persisted in all the categories except concentrates. In the case of juices, a monthly increase in affordability is observable in the post-versus pre-tax period. Concentrates showed an important change in the direction of the post-tax trend, rapidly reducing their affordability after the tax policy entered into effect.

Our results confirmed that, upon tax implementation, there was a downward change in the affordability index at least for carbonates, concentrates and bottled water (Table 2). There was a 5.77% (CI 95%–7.80%; −3.73) reduction in the affordability of carbonates. While, for the concentrate group, similar to the price analysis, no level effects were detectable immediately after the policy, but a lagged effect is evident in the post-tax period, with a reduction of 0.96% (CI 95% −1.80; −0.12%) per month compared with the long-term trend for this category. Consistent with the price analysis, juices and energy and sport drinks showed no consistent effects on affordability resulting from the policy.

**Table 2:** Affordability of soft drinks (% change) after the taxation implemented in Chile.

	Carbonates		Juices		Concentrates		Bottled water		Energy and sport drinks	
	Model 1	Model 2	Model 1	Model 2	Model 1	Model 2	Model 1	Model 2	Model 1	Model 2
Implementation effect	−4.23*	−5.77*	−2.02*	−0.78	0.42	0.70	−3.71*	−2.67*	−2.89*	−1.18
	[-5.78; −2.67]	[-7.80; −3.73]	[ −3.71; −0.70]	[-2.38; 0.83]	[-2.25; 3.08]	[-3.15; 4.54]	[-5.13; −2.28]	[-4.44; −0.90]	[-5.33; −0.45]	[-4.73; 2.36]
Announcement effect		−1.14		1.87*		0.27		0.53		1.63
		[-2.59; 0.31]		[0.33; 3.41]		[-2.41; 2.94]		[-0.99; 2.05]		[-0.86; 4.12]
General time trend (monthly)	0.08	0.05	−0.08	−0.09	0.05	0.05	−0.15*	0.00	0.63	0.53
	[-0.16; 0.32]	[-0.13; 0.23]	[-0.27; 0.10]	[-0.28; 0.10]	[-0.46; 0.56]	[-0.46; 0.56]	[-0.21; −0.09]	[-0.21; 0.21]	[-0.02; 1.29]	[-0.12; 1.19]
Post-tax time trend (monthly)	−0.17	−0.18*	0.17*	0.22*	−0.96*	−0.96*	0.10*	0.11	0.03	0.11
	[-0.52; 0.18]	[-0.33; −0.04]	[0.03; 0.30]	[0.12; 0.31]	[-1.80; −0.12]	[-1.80; −0.12]	[0.00; 0.19]	[-0.00; 0.22]	[-0.70; 0.76]	[-0.61; 0.84]
Selected ARIMA model	(2,1,2)	(2,1,1)	(1,0,1)	(1,0,0)	(1,1,0)	(1,1,0)	(2,0,0)	(1,0,0)	(0,1,0)	(0,1,0)

Model 1: ARIMAX model without announcement effect. Model 2: ARIMAX model with announcement effect. Point estimates in percent change. 95% Confidence Intervals in parenthesis. *p < 0.05.

### 3.3 Sensitivity analysis

In order to examine potential structural uncertainty underlying the model selection, we tested alternative methodological approaches and, overall, our results were robust. First, we conducted an exploratory analysis for sudden changes in the price series (See Supporting Information – SI3 Placebo test). This analysis confirmed for the carbonates and juice categories that an unexpected change occurred in October 2014, concordant with the hypothesis that an exogenous event affected the overall trends. Moreover, using different estimation methods, the effect in prices found were consistent with our main results based on the ARIMAX model (See Supporting Information – SI5 Sensitivity analysis). Also, concordantly to our model estimates, prices of concentrates showed an increase after the implementation of the tax, with a particularly sharp increase in December 2014. Interestingly, the outlier explorations suggest that the post-tax increase in prices of waters exhibited a decreasing trend, nearly converging with pre-tax price trajectories.

Secondly, alternative regression methods for interrupted time series analysis using non-related products (i.e. detergents) as a comparison group were implemented (See Supporting Information – SI5 - Sensitivity analysis). The results obtained largely confirmed our ARIMAX effect estimates. For the categories in which significant effects were found, the magnitude of the price level change was even larger when we used panel-data estimation methods, particularly fixed-effect models for each product: prices of carbonates were found to have increased by 8.46% (CI 95% 6.12; 10.79%) after the tax implementation. In the juice category, all alternative models pointed to an increase of nearly 3% in prices post-implementation, similar to the effect predicted by the ARIMAX model without the inclusion of the announcement effect indicator variable (See Supporting information – SI5 - Sensitivity analysis). Estimates from models adjusting by food inflation showed similar results (See Supporting information – SI5 - Sensitivity analysis).

## 4 Discussion

To our knowledge, this is the first study to evaluate the effects on market prices and consumer's affordability of soft drinks at the population level, comparing pre and post-tax time trends. We used nationally representative, official price data from Chile, analysed using ARIMA models with exogenous variables to estimate the policy effects based on the time-series of different soft drink categories. After adjustment for time-trends, inflation and external economic shocks; our analysis shows changes in prices attributable to the implementation of the policy.

Considering that the expected average net price effect of the tax for carbonates assuming a 100% pass-through was 4%, the observed 5.6% price increase in this product category represents an over-shifting (140%). These results were robust to different empirical estimation approaches. Reports by main carbonates manufacturers largely confirm that the industry increased its prices in response to the tax (Guerrero, 2014) and that there were negative effects on sales volumes (Coca-Cola Andina, 2014; Coca-Cola Embonor, 2014). In contrast, considering an expected price increase of 3.8% and 18% in the juice and energy and sport drinks categories respectively, no consistent effects in prices or affordability were detected in our analysis. For the products that were affected by tax reductions, such as concentrates and bottled water, no or negative effects were observed with increasing prices concomitant – or just subsequent after – the policy implementation. The latter is the case for concentrates, a group largely affected by an overall tax reduction, were we expected a net average price reduction of 2.8%; nevertheless, increasing prices were observed in the post-tax period for this product category. Moreover, a discrete increasing effect in water prices was observed after the tax. The vast majority of bottled water were not affected by the tax increase (i.e. plain water) or obtained a tax reduction (i.e. flavoured waters), with an expected overall average net price reduction of 1.2%. This counter-intuitive phenomenon may be explained by a strategic response from the industry, or other market factors such as increasing demand due to substitutional effects.

The pass-through of soft drink taxes has been found to be highly variable across countries and states that had implemented such policies. Evidence from the tax in Berkeley, California showed a modest pass-through of the tax – only 27.1% (Cawley and Frisvold, 2015) or 47% (Falbe et al., 2015) of the tax change were passed to the consumer prices (under-shifting). In contrast, in the case of Mexico, where the expected price increase after the tax would have been approximately 9%, it was found that prices raised by 12% (over-shifting) (Grogger, 2015). Such heterogeneity across findings could be explained by differences in the magnitude of the tax, the scale of implementation (national versus city), the demand elasticity, and market characteristics, among other relevant factors. The degree of pass-through across various beverages may also be partly due to the portfolio of firms. Many firms produce a mixture of high-, low-, and un-taxed beverages, so they can cost-shift strategically.

Our results differ in some ways compared with two recent studies (Caro et al., 2018; Nakamura et al., 2018), one of which was conducted by ourselves, that have analysed this tax-reform in Chile using household purchase data. For the group of beverages that were unaffected by tax changes, Nakamura et al. found a non-significant increase of 1.7% (SE 0.02), while Caro et al. reported a significant increase in the price of 1.8% (CI 95% 0.7; 2.9%). Both studies are consistent with our results, and even though the size of the effect differs, the confidence intervals of the estimations overlap.

For beverages subject to a tax increase, Caro et al. found a significant 2.0% (CI 95% 1.0; 3.0%) and 3.9% (CI 95% 1.6; 6.2%) increase in the prices for carbonates and non-carbonates drinks, respectively. The study of Nakamura et al. found a significant increase of 1.9% (SE 0.01) in the price of the high-tax products associated with the announcement of the policy, while no significant effect was observed at the time of implementation of the tax policy. Moreover, the latter study does not provide aggregated information for carbonates and non-carbonates drinks. Our results for carbonates are concordant with the findings of Caro et al. in terms of the direction of the change; however, we found a higher magnitude of the effect. In the case of juices, which would correspond to the category of non-carbonated drinks in Caro et al. our results differ since we did not find consistent evidence of a price increase.

For the low-sugar soft drinks, Nakamura et al. found a significant 1.7% (SE 0.01) decrease in the price after the implementation of the tax reform. In the case of Caro et al. the authors found a non-significant change for ready-to-drink beverages and a significant decrease of 6.7% (CI 95% 4.6; 8.2%) for concentrates. Our results are different compared with both studies, since we found a significant increase in the price for concentrates.

Compared to the aforementioned studies, our analysis has both strengths and limitations that could drive the observed differences. The main strength is that we use data that contains information from posted prices in stores, while Nakamura et al. and Caro et al. used data from take-home purchases. Compared to the prices collected from purchase records, the data obtained directly from stores are a more reliable source of information and are less prone to bias. This is because if consumers do not buy products that become more expensive after the tax increase, the price estimations from purchase data would underestimate the real effect of the price changes due to searching behaviours or shifts towards cheaper alternative brands. The opposite could occur in the case of the products affected by a tax decrease. Moreover, take-home purchase data could suffer from measurement error because consumers may not always report purchases accurately.

Our study has several limitations. First, as was already mentioned, in our data it was not possible to discriminate the tax category for each product, which is a limitation compared to the data used by the two recent impact studies. Therefore, our observed effects on carbonates could mask differences in subgroup responses. The price of regular sodas (affected by a tax increment) may have risen more than the price of their diet versions (affected by a tax reduction), thus underestimating the true effect on soda products that were affected by the tax increase. This limitation implies that our absolute price effect estimates are likely conservative. Nevertheless, we still found a larger effect than in previous studies. To account for this potential problem, we use a reference expected price change based on the market share of low and high sugar products within each category. Secondly, due to the characteristic of the implemented policy, the estimates rely on an observational study design in which unobservable confounders could not be completely controlled. To address this problem, we implemented models to account for before and after variations. Alternatively, we used non-related products as control groups to test the robustness of our results. Thirdly, in other contexts, an increase in distributors’ margins was reported after changes in soft drink taxes (ECORYS, 2014). This is an alternative explanation to the observed price response that we could not account for within our analysis. Fourthly, our main outcomes of interest in this study were only intermediate outcomes for the decision to purchase and consume soft drinks. Both prices and affordability are important factors that could increase or decrease consumption, but in isolation they cannot account for the complexity of the dietary behaviours problems within the population.

### 4.1 Policy implications

We found an increasing trend for soft drinks affordability prior to the tax change, which is in line with recent findings in the international literature (E. Blecher et al., 2017). Following the implementation of the tax, a decrease in affordability was observed in some categories. However, the secular upward trend in affordability has continued for all soft drinks, except of concentrates, at least in the medium term after the tax implementation. Our finding highlights the importance of designing fiscal policies that could take into account changes in the population's purchasing power. In the medium to long-term, assuming that the relative prices of other goods remain constant, a fiscal policy that is not dynamically adjusted could become ineffective at decreasing the affordability of these products, particularly in contexts of rapid growth of purchasing power as is observed in emerging economies. This could be particularly relevant for excise taxes that are not indexed to relevant macroeconomic variables as inflation or wages, as is the case in both the Berkeley and Mexico taxes. In our study, we found important heterogeneity in terms of the price response and pass-through across product categories. While carbonates and powdered juices showed a price response after the tax implementation, no clear effects were identified for juices and energy drinks. Similar to our findings, the tax implemented in Hungary showed under-shifting or negative pass-through for juices and energy drinks, while complete pass-through was observed for other soft drink categories including carbonates (ECORYS, 2014). In France, the pass-through was higher for SSB (53%) than for non-calorically sweetened beverages (37%), and lower for top national brands (22%) compared to other brands (52%–86%) (Etilé et al., 2018). In Denmark, the results of a study of the impact of a soft drink excise tax exhibited important levels of heterogeneity across products. Mainly over-shifting was observed among products that were subject to a tax increase, whereas under-shifting was the predominant effect among products that experienced a tax cut (ECORYS, 2014). In a context of simultaneous mixed tax changes, our findings confirm both heterogeneity of price response to tax increases and under-shifting to tax reductions. This is something that decision makers designing a soft drink tax should consider cautiously. These findings highlight the importance of conducting impact evaluations of fiscal policies differentiating the effects by soft drink product categories, rather than overall estimates across all soft drinks. If the price and pass-through effects are category specific, we would expect that any potential effect in terms of consumption would also be mixed and, hence, difficult to predict in terms of its overall net effect.

Some considerations regarding the Chilean market and the implementation of the tax are worth noting to interpret our findings from a policy perspective. In Chile, two manufacturers held 82% of the soft drinks market-share during 2014 (Euromonitor, 2016). In a context of a highly concentrated market, a policy design with a simultaneous increase and decrease of tax rate to different non-alcoholic beverages, the response of the industry could be a factor that could potentially undermine the expected effects of the policy. Since any given manufacturer produces different beverage categories, both above and below the tax threshold, a strategic response from the industry is likely to occur. This regulatory design could threaten the desired effect of the policy, since the tax revenue is not levied on the distribution chain but at the producers' level, and companies can mitigate the tax effect on prices by spreading the effect in all their products. Furthermore, an important implementation aspect of the Chilean tax to consider is the reliance on industry's self-reports. Periodically, manufacturers are compelled to report the total sales and values of products in each broad tax category (above and below the 6.25 sugar grs per 100 ml threshold). Based on these reports and without any formal mechanism to audit the data provided, the Chilean Internal Revenue Office estimates the tax burden to be paid by the manufacturers in each period. This soft-regulation strategy could lead to an opportunistic response from the industry, attempting to mitigate the effectiveness of the policy. Another important aspect of the two-tiered design of the Chilean tax is that it could become a strong incentive for reformulation. Nevertheless, available data does not allow the direct evaluation of the impact of the tax on reformulation. In the case where this a high level of reformulation induced by the policy, we expect that there would be a reduction in the prices soft drink product reformulated, due to the 8% points difference between low and high sugar products. Hence, the overall price effect of the policy would be lessened mitigated in such case.

## 5 Conclusion

Due to the likely contribution of soft drink consumption to obesity and other non-communicable disease, several countries are implementing new policies to cut down their consumption, such as via soft drink taxes. Learning from the policy experiences of other countries has the potential to provide relevant evidence for policymakers considering interventions. The Chilean experience showcases that a mixed tax modification, affecting differentially high sugar and low sugar soft drinks, produced changes in both market prices and affordability of taxed products. Nevertheless, effects were heterogeneous across product categories, and sometimes different from what might have been expected. Both policy design and market structure could be potential explanations for this phenomenon and should be carefully considered by policymakers before implementation of a similar tax in other settings.

## Contributions

Conceptualization: CC, JD, NS, AM, RN, MS, Methodology: CC, JD, NS, Software: CC, JD, Validation: CC, NS, Formal analysis: CC, Investigation: CC, Data curation: CC, JD, Writing – original draft preparation: CC, NS, JD, Writing - review and editing: CC, JD, NS, AM, RN, MS, Visualization: CC, Supervision: CC, MS, Funding acquisition: CC, NS, AM, RN, MS

## Declarations

Ethics approval and consent to participate: Ethical approval was not required for this study due to the aggregate and public nature of the data used.

Consent for publication: All authors declare to have approved the final version of the manuscript and consent its publication.

Availability of data and material: Datasets and R scripts are available upon request to the authors.

Competing interests: Authors declare no conflicts of interest related to the content of this article.
