# Supplementary material for: Effects of a sugar-sweetened beverage tax on prices and affordability of soft drinks in Chile: A time series analysis
Source: Soc Sci Med. 2020 Jan;245:112708. doi: 10.1016/j.socscimed.2019.112708 (PMC7267770; doi:10.1016/j.socscimed.2019.112708)
Supplement: Multimedia component 1 [file mmc1.docx]

Supporting information

**Effects of a Sugar-Sweetened Beverage Tax on Prices and Affordability of Soft Drinks in Chile**

Table of contents

[SI1 - Market share and expected price increase by product category 3](#_Toc24739843)

[SI2 - Model diagnostics 4](#_Toc24739844)

[Test for unit-root 4](#_Toc24739845)

[Test for autocorrelation 5](#_Toc24739846)

[SI3 - Placebo test 6](#_Toc24739847)

[Effects in non-taxed unrelated products 6](#_Toc24739848)

[Exploring unexpected breaks in the time-series 7](#_Toc24739849)

[SI4 - Graphical representation of ARIMA model results 13](#_Toc24739850)

[SI5 - Sensitivity analysis 17](#_Toc24739851)

[Analysis adjusted by food inflation 17](#_Toc24739852)

[Before and after design without a control group 19](#_Toc24739853)

[Alternative methods for impact effect estimation 19](#_Toc24739854)

[Before-after with control group design 20](#_Toc24739855)

[Interrupted Time-Series Analysis with control group 20](#_Toc24739856)

[References 28](#_Toc24739857)

*Table S1: Share of volume purchases share (volume) by product an tax category in Chile 2011-2015* 3

*Table S2: Expected price change for each product category after the implementation of the policy.* 3

*Table S3: Results from Augmented Dickey-Fuller Test for unit-root for price models* 4

*Table S4: Results from Augmented Dickey-Fuller Test for unit-root for affordability models* 4

*Table S5: Results Ljung-Box Test for autocorrelation for price models* 5

*Table S6: Results Ljung-Box Test for autocorrelation for affordability models* 5

*Table S7: Price effect (% change) on non-taxed products before and after-tax reform (ARIMAX models)* 6

Table S8: Price effect (% change) on soft drink after the taxation implemented in Chile, adjusted by food inflation 18

Table S9: Price effect (% change) of soft drink taxation implemented in Chile (AR1 models) 19

Figure S1: Results of outlier detection (level and temporary effect estimation) for carbonates 8

Figure S2: Results of outlier detection (level and temporary effect estimation) for bottled water 9

Figure S3: Results of outlier detection (level and temporary effect estimation) for juices 10

Figure S4: Results of outlier detection (level and temporary effect estimation) for concentrates 11

Figure S5: Results of outlier detection (level and temporary effect estimation) for energy drinks 12

Figure S6: Observed and predicted counterfactual trends for carbonates (ARIMA) 13

Figure S7: Observed and predicted counterfactual trends for bottled water (ARIMA) 14

Figure S8: Observed and predicted counterfactual trends for juices (ARIMA) 15

Figure S9: Observed and predicted counterfactual trends for concentrates (ARIMA) 16

Figure S10: Price effect (% change) on carbonates compared to controls after the taxation implemented in Chile 21

Figure S11: Affordability effect (% change) on carbonates compared to controls after the taxation implemented in Chile 22

Figure S12: Price effect (% change) on juice compared to controls after the taxation implemented in Chile 23

Figure S13: Affordability effect (% change) on juice compared to controls after the taxation implemented in Chile 24

Figure S14: Price effect (% change) on concentrates compared to controls after the taxation implemented in Chile 25

Figure S15: Affordability effect (% change) on concentrates compared to controls after the taxation implemented in Chile 26

Figure S16: Price effect (% change) on water compared to controls after the taxation implemented in Chile 27

Figure S16: Affordability effect (% change) on water compared to controls after the taxation implemented in Chile 28

# SI1 - Market share and expected price increase by product category

*Table S1: Share of volume purchases share (volume) by product an tax category in Chile 2011-2015*

| **Product category** | Proportion | **No tax** | **10% tax** | **18% tax** | **Total** |
| --- | --- | --- | --- | --- | --- |
| *Carbonates* | 53.1% | 0% | 12.1% | 87.9% | 100.0% |
| *Concentrates* | 22.6% | 0% | 98.1% | 1.93% | 100.0% |
| *Juice* | 16.1% | 0% | 14.4% | 85.6% | 100.0% |
| *Bottled water* | 8.1% | 58.6% | 41.0% | 0.4% | 100.0% |
| Total | 100.0% | 4.8% | 34.3% | 61.0% | 100.0% |

Source: Own estimations based on Kantar World Panel data Chile - January 2011 to December 2015. No data for sports and energy drinks was available.

Products in the 10% tax category were expected to reduce prices in 3% and products in the 18% tax rate to increase in 5%. To have a reference value of the expected average effect of the tax reform in each product category, we estimated a weighted average based on the market share presented in table 1. No data for sports and energy drinks data was available. Nevertheless, this category had a 0% rate before the tax, therefore all products were subject to a 10 or 18% net increase in the tax rate.

*Table S2: Expected price change for each product category after the implementation of the policy.*

| **Product category** | **Expected price change** |
| --- | --- |
| *Carbonates* | 4.0% |
| *Concentrates* | -2.8% |
| *Juice* | 3.8% |
| *Bottled water* | -1.2% |
| *Sports and energy drink* | 18%* |

* Most conservative estimate

# SI2 - Model diagnostics

## Test for unit-root

To assess the stationarity of the time-series analysed, an Augmented Dickey-Fuller Test for unit-root was performed. In the series in which the test did not reject the null hypothesis of non-stationarity, the first difference of the series was estimated, and a new test performed to confirm the stationarity. Results for the test is presented in table S3 and S4

*Table S3: Results from Augmented Dickey-Fuller Test for unit-root for price models*

| Univariate price series | Non-differenced series | | First difference | |
| --- | --- | --- | --- | --- |
|  | DF | p-value |  | p-value |
| *Carbonates* | -2.4024 | 0.4103 | -10.754 | <0.01* |
| *Concentrates* | -1.2278 | 0.8948 | -7.1753 | <0.01* |
| *Juice* | -3.9474 | 0.01522* | - | - |
| *Bottled water* | -3.5073 | 0.04565* | - | - |
| *Sports and energy drink* | -3.9353 | 0.02097* | - | - |
| *Milk* | -1.6379 | 0.7256 | -6.466 | <0.01* |
| *Laundry detergent* | -3.165 | 0.09833 | -12.344 | <0.01* |
| *Dish detergent* | -1.8347 | 0.6445 | -6.7447 | <0.01* |
| *Napkin and toilet papers* | -2.6474 | 0.3093 | -11.479 | <0.01* |

* Significant at a p=0.05

*Table S4: Results from Augmented Dickey-Fuller Test for unit-root for affordability models*

| Univariate price series | Non-differenced series | | First difference | |
| --- | --- | --- | --- | --- |
|  | DF | p-value |  | p-value |
| *Carbonates* | -3.2055 | 0.09175 | -11.785 | <0.01* |
| *Concentrates* | -1.4875 | 0.7875 | -7.2683 | <0.01* |
| *Juice* | -4.9789 | <0.01* | - | - |
| *Bottled water* | -4.626 | <0.01* | - | - |
| *Sports and energy drink* | -3.2724 | 0.08858 | -6.126 | <0.01* |
| *Milk* | -1.9753 | 0.5865 | -6.8802 | <0.01* |
| *Laundry detergent* | -1.959 | 0.5931 | -7.0029 | <0.01* |
| *Dish detergent* | -3.6042 | 0.0372* | - | - |
| *Napkin and toilet papers* | -3.387 | 0.06177 | -11.654 | <0.01* |

* Significant at a p=0.05

## Test for autocorrelation

To assess that the selected ARIMA models adequately accounted for the autocorrelation of the series, a Ljung-Box Test for serial autocorrelation for the model residuals was performed. In case that the test failed to reject the null hypothesis of no autocorrelation on the residuals, a new ARIMA model was fitted. Results for the Ljung-Box test for our selected models are presented in tables S5 and S6

*Table S5: Results Ljung-Box Test for autocorrelation for price models*

| Univariate price series | Model 1 (without announcement effect | | | Model 2 (with announcement effect) | | |
| --- | --- | --- | --- | --- | --- | --- |
|  | ARIMA | χ2 | p-value | ARIMA | χ2 | p-value |
| *Carbonates* | (1,1,0) | 18.123 | 0.112 | (1,1,0) | 16.39 | 0.174 |
| *Concentrates* | (0,1,1) | 9.0032 | 0.7027 | (0,1,1) | 7.5651 | 0.8181 |
| *Juice* | (1,0,1) | 9.2149 | 0.6845 | (1,0,0) | 10.121 | 0.6053 |
| *Bottled water* | (1,0,0) | 9.73 | 0.6396 | (1,0,0) | 9.0244 | 0.7008 |
| *Sports and energy drink* | (1,0,0) | 17.329 | 0.1376 | (1,0,0) | 18.732 | 0.09521 |
| *Milk* | (3,1,2) | 16.411 | 0.1731 | (3,1,2) | 14.718 | 0.2572 |
| *Laundry detergent* | (0,1,1) | 12.776 | 0.3855 | (0,1,1) | 12.732 | 0.3888 |
| *Dish detergent* | (1,1,0) | 9.9091 | 0.6239 | (1,1,0) | 10.065 | 0.6102 |
| *Napkin and toilet papers* | (1,1,1) | 4.2135 | 0.9793 | (1,1,1) | 4.4 | 0.9751 |

*Table S6: Results Ljung-Box Test for autocorrelation for affordability models*

| Univariate price series | Model 1 (without announcement effect | | | Model 2 (with announcement effect) | | |
| --- | --- | --- | --- | --- | --- | --- |
|  | ARIMA | χ2 | p-value | ARIMA | χ2 | p-value |
| *Carbonates* | (2,1,2) | 8.7534 | 0.7238 | (2,1,1) | 16.979 | 0.1504 |
| *Concentrates* | (1,1,0) | 10.178 | 0.6003 | (1,1,0) | 10.425 | 0.5787 |
| *Juice* | (1,0,1) | 9.4778 | 0.6617 | (1,0,0) | 9.909 | 0.6239 |
| *Bottled water* | (2,0,0) | 8.9439 | 0.7077 | (1,0,0) | 9.5099 | 0.6589 |
| *Sports and energy drink* | (0,1,0) | 8.4228 | 0.5876 | (0,1,0) | 8.8406 | 0.5473 |

# SI3 - Placebo test

## Effects in non-taxed unrelated products

To account for potential external events others than the SSB taxation (i.e. economic cycles; other fiscal policies implemented concomitantly) that could alternatively explain the observed price effects, we tested similar ARIMAX model on other product categories that were expected to be unrelated to the implemented policy (detergents, napkins and toilet papers) and potential substitutes not directly affected by the tax modification (milk). As expected, no consistent price effects were detected in these products after the implementation of the tax on SSB, thus suggesting that the effects observed in the SSB category are not attributable to other potential concomitant economic shock.

*Table S7: Price effect (% change) on non-taxed products before and after-tax reform (ARIMAX models)*

|  | Milk | | Laundry detergents | | Dish detergents | | Napkin and toilet papers | |
| --- | --- | --- | --- | --- | --- | --- | --- | --- |
| Implementation effect | **-3.36*** | -0.82 | -0.49 | 0.98 | 0.15 | -0.49 | -1.53 | -1.74 |
|  | [-5.08; -1.63] | [-3.96; 2.32] | [-4.35; 3.39] | [-4.52; 6.48] | [-2.34; 2.63] | [-3.98; 3.00] | [-3.87; 0.82] | [-5.01; 1.64] |
| Announcement effect |  | 1.59 |  | 1.45 |  | -0.63 |  | -0.17 |
|  |  | [-0.07; 3.25] |  | [-2.44; 5.35] |  | [-3.01; 1.80] |  | [-2.49; 2.13] |
| Time trend (monthly) | **0.59*** | **0.65*** | 0.03 | -0.02 | 0.30 | 0.31 | -0.03 | -0.03 |
|  | [0.09; 1.09] | [0.15; 1.15] | [-0.86; 0.90] | [-0.86; 0.90] | [-0.05; 0.65] | [-0.05; 0.66] | [-0.40; 0.34] | [-0.40; 0.34] |
| Post-tax trend (monthly) | -0.03 | -0.08 | 0.45 | 0.46 | 0.28 | 0.27 | 0.37 | 0.37 |
|  | [-0.98; 0.92] | [-1.01; -0.87] | [-1.08; 1.98] | [-1.06; 1.99] | [-0.17; 0.73] | [-0.18; 0.72] | [-0.17; 0.91] | [-0.17; 0.91] |
| Selected ARIMA model | (3,1,2) | (3,1,2) | (1,1,0) | (1,1,0) | (0,1,1) | (0,1,1) | (1,1,1) | (1,1,1) |
| Model 1: ARIMAX model without announcement effect. Model 2: ARIMAX model with announcement effect. Point estimates in percent change. 95% Confidence Intervals in parenthesis. *p<0.05 | | | | | | | | |

## Exploring unexpected breaks in the time-series

Transitory changes are frequent in time-series data due to random variation. Moreover, even changes produced by exogenous factor could the only transitory, making the effects of the exogenous treatment (i.e. a fiscal policy) be negligible in the long-term forecast. Chen and Liu proposed a methods for outlier detection based on unexpected breaks in the time-series data: level shifts - unexpected changes in the mean level of the prices- and temporary changes – level changes with a posterior tendency to converge with the long term forecast (1). Based on the automatized procedure implemented in the R package tsoutliers(2), is possible to fit ARIMA models and detect outliers without needing to pre-specify any hypothesis of potential exogenous effect in the series. Therefore, this exploratory method allows to confirm: a) If there are level outliers in periods of the time-series were exogenous interventions or events had been known to had occur (i.e. announcement or enforcement of a new policy); b) If the observed changes in the series correspond to level changes in the outcome or are transient modifications that later converge in the same previous trend (temporary changes); and c) to explore if there are any other potential unexpected breaks in the data that are not explained by known exogenous factors (i.e. changes on data quality, unknown interventions).

Graphical representation of level shifts and temporary changes in the time-series for each product category is presented in Figure 1 to 5. In the upper plot the expected trend without the effect of the outlier is presented in red (counterfactual), meanwhile the observed trend is represented in green. The estimated cumulative effect of change in prices (in percentage) is presented in the lower part of each series with a black line.

Figure S1: Results of outlier detection (level and temporary effect estimation) for carbonates


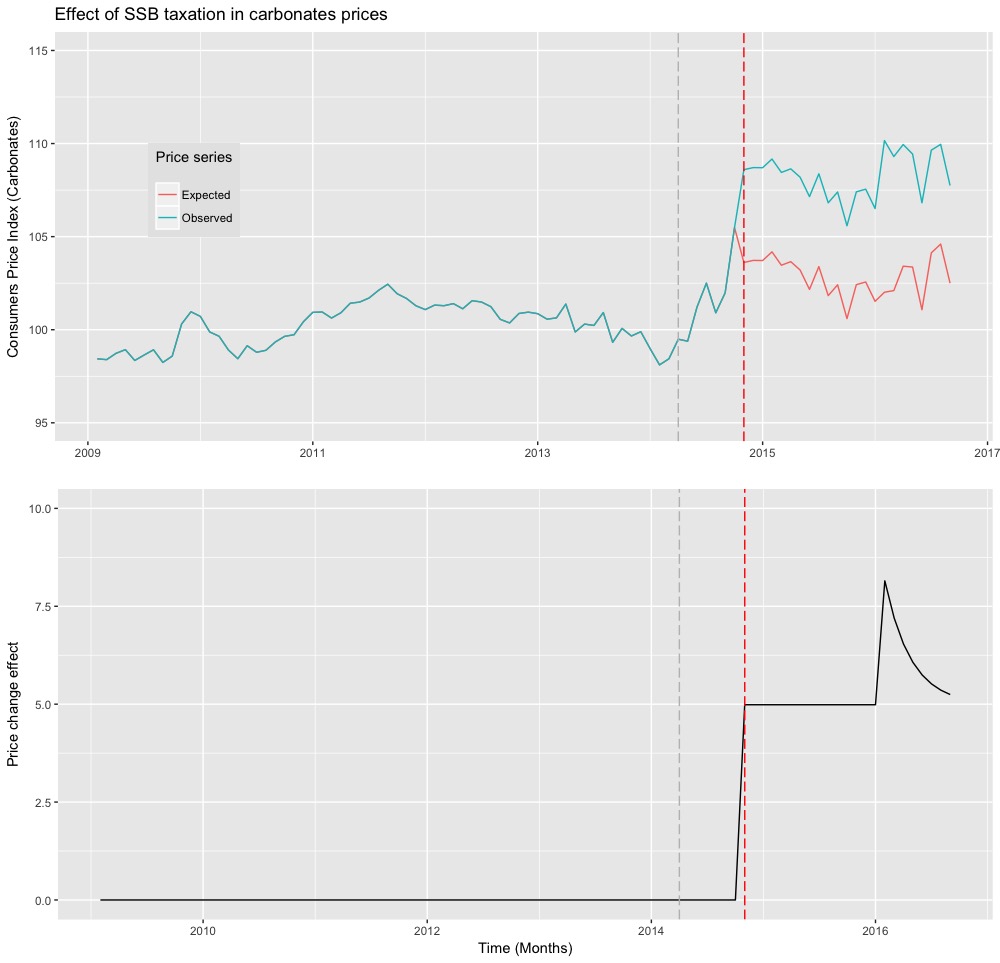


Figure S2: Results of outlier detection (level and temporary effect estimation) for bottled water


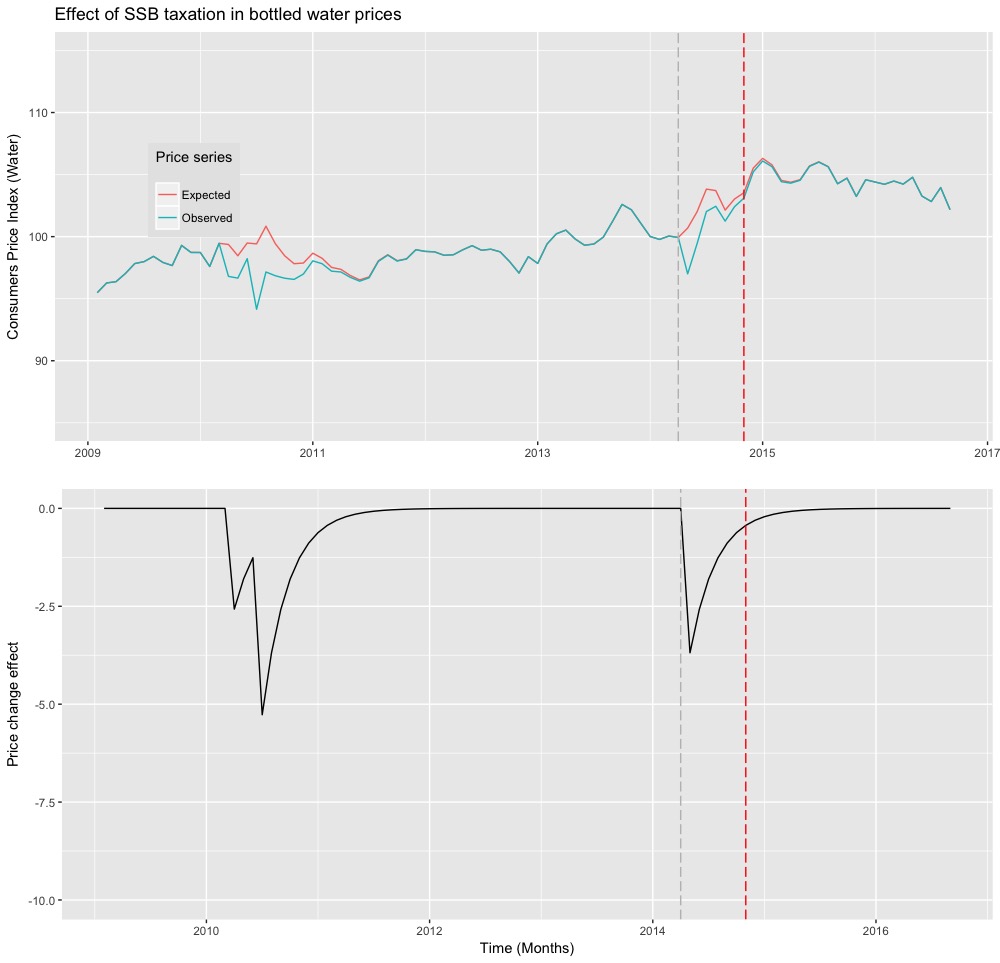


Figure S3: Results of outlier detection (level and temporary effect estimation) for juices


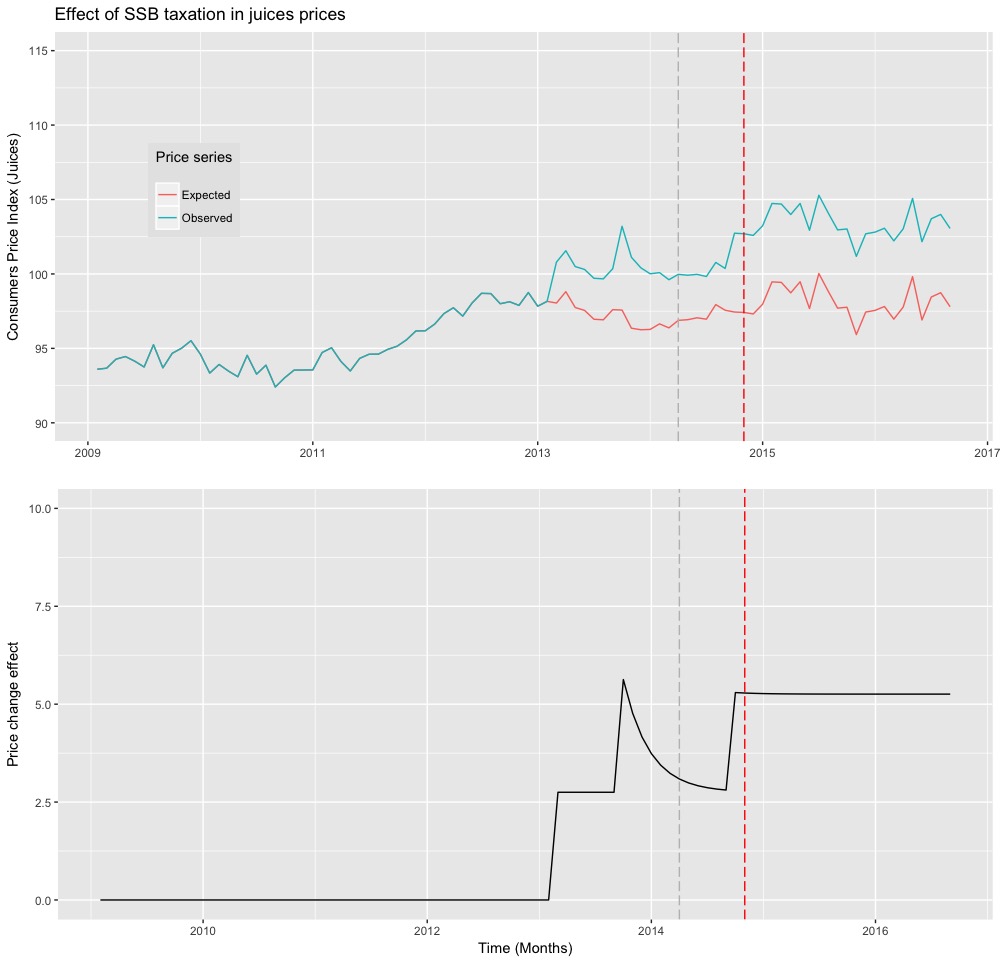


Figure S4: Results of outlier detection (level and temporary effect estimation) for concentrates


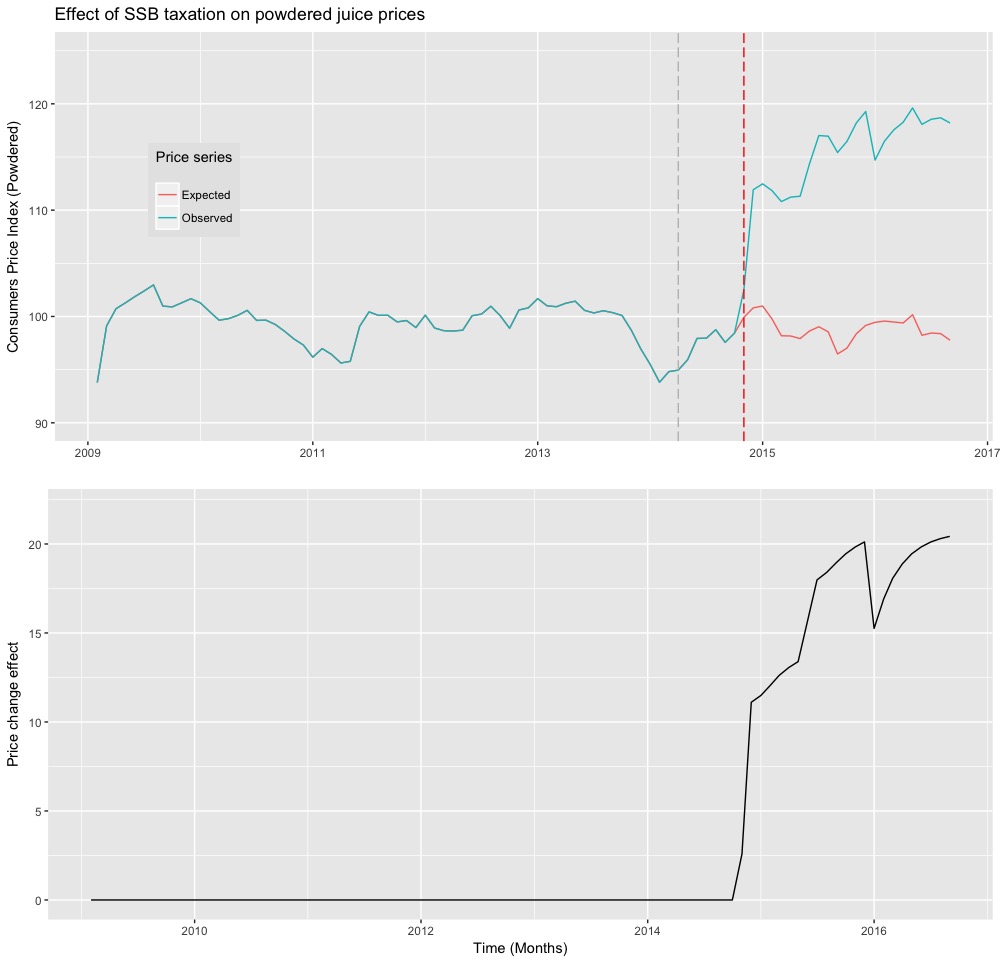


From November 2014 several level shifts were detected in a stepped process. The most important in term of magnitude occurs in December 2014 (14,1% increase). With a cumulative effect near to 20% at the end of the observed series. These findings are concordant to what is found in ARIMAX and ITSA models, were the maximum effect in prices is observed with a lag of 2-3 months after the implementation of the policy.

Figure S5: Results of outlier detection (level and temporary effect estimation) for energy drinks


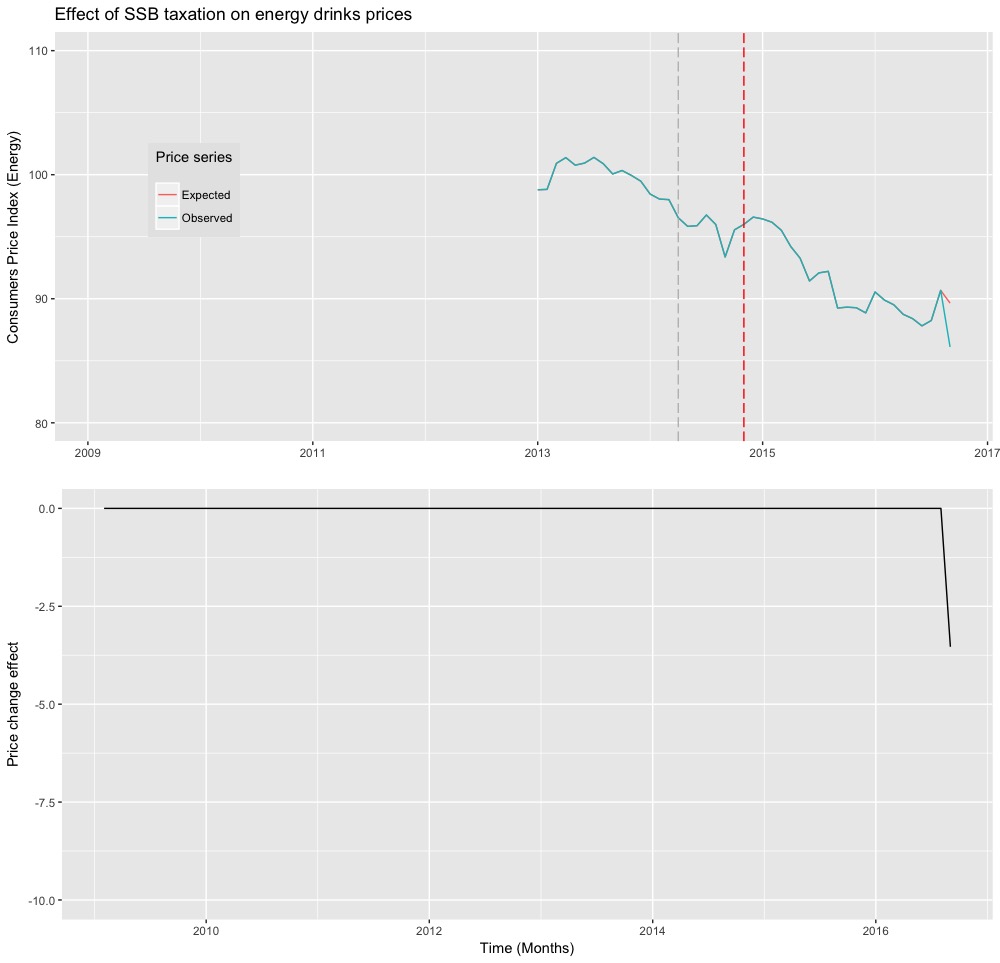


# SI4 - Graphical representation of ARIMA model results

In this section we explore the graphical results for the model prediction for each product univariate time-series. First, an ARIMA model including exogenous variables (see Methods) was estimated using Box-Jenkins approach (See Methods section in the main manuscript for full description of the methods) (3). The predicted values of this model are presented in oranges lines contrasted with the observed data (blue circles), demonstrating the high capacity of the fitted model to capture the variability and trends of the series. Secondly, an ARIMA model based only in the observed data before the announcement of the policy was fitted. This model was used to forecast price trends for the period February 2014 onwards. Predicted values of this model are presented in green line, showing a potential counterfactual trend based on the previous price data. Therefore, differences between orange and green line could be attributed to exogenous effects such as the SSB tax policy implemented in October 2014. In the case of carbonates and powder juices the differences between the series is evident early after the policy implementation (red dotted line). For bottled waters, differences are graphically evident but tend to fade out converging again in the long-term (2016).

Figure S6: Observed and predicted counterfactual trends for carbonates (ARIMA)

Grey dotted line: policy announcement. Red dotted line: policy implementation.

Figure S7: Observed and predicted counterfactual trends for bottled water (ARIMA)

Grey dotted line: policy announcement. Red dotted line: policy implementation.

Figure S8: Observed and predicted counterfactual trends for juices (ARIMA)

Grey dotted line: policy announcement. Red dotted line: policy implementation.

Figure S9: Observed and predicted counterfactual trends for concentrates (ARIMA)

Grey dotted line: policy announcement. Red dotted line: policy implementation.

# SI5 - Sensitivity analysis

## Analysis adjusted by food inflation

As a sensitivity analysis, we estimated the same ARIMAX models but including as a control variable the food inflation to adjust for specific changes over the price foods. Results are presented in table S8 below and confirm that our conclusions are not sensitivity to this different specification for the model.

Table S8: Price effect (% change) on soft drink after the taxation implemented in Chile, adjusted by food inflation

|  |  | Carbonates | | Juices | | Concentrates | | Bottled water | | Energy and sport drinks | |
| --- | --- | --- | --- | --- | --- | --- | --- | --- | --- | --- | --- |
|  |  | Model 1 | Model 2 | Model 1 | Model 2 | Model 1 | Model 2 | Model 1 | Model 2 | Model 1 | Model 2 |
| Implementation effect |  | **5.18*** | **6.52*** | **2.16*** | 0.80 | 1.28 | 1.24 | **3.02*** | **2.51*** | 2.30 | 0.36 |
|  |  | [3.40; 6.97] | [4.10; 8.94] | [0.49; 3.82] | [-1.04; 2.66] | [-1.94; 4.50] | [-3.15; 5.63] | [1.36; 4.71] | [0.65; 4.37] | [-0.01; 4.61] | [-2.95; 3.68] |
| Announcement effect |  |  | 1.26 |  | **-1.85*** |  | -0.04 |  | -0.92 |  | -1.70 |
|  |  |  | [-0.34; 2.86] |  | [-3.59; -0.11] |  | [-3.00; 2.92] |  | [-2.56; 0.72] |  | [-3.79; 0.37] |
| Time trend (monthly) |  | 0.04 | 0.02 | 0.23 | 0.21 | 0.13 | 0.13 | 0.03 | 0.00 | **-0.51*** | -0.42 |
|  |  | [-0.27; 0.34] | [-0.27; 0.32] | [-0.07; 0.52] | [-0.07; 0.50] | [-0.50; 0.76] | [-0.50; 0.76] | [-0.28; 0.35] | [-0.30; 0.32] | [-0.96; -0.05] | [-0.87; 0.04] |
| Post-tax trend (monthly) |  | 0.15 | 0.17 | **-0.21** | **-0.24** | **0.99*** | **0.99*** | **-0.14*** | **-0.16*** | -0.04 | -0.14 |
|  |  | [-0.17; 0.47] | [-0.14; 0.48] | [-0.34; -0.08] | [-0.35; -0.14] | [0.08; 1.87] | [0.06; 1.90] | [-0.25; -0.02] | [-0.27; -0.05] | [-0.43; 0.35] | [-0.49; 0.22] |
| Selected ARIMA model |  | (1,1,0) | (1,1,0) | (1,0,1) | (1,0,0) | (0,1,1) | (0,1,1) | (1,0,0) | (1,0,0) | (1,0,0) | (1,0,0) |

Model 1: ARIMAX model without announcement effect. Model 2: ARIMAX model with announcement effect. Point estimates in percent change. 95% Confidence Intervals in parenthesis. *p<0.05. ^♦^ Expected change in prices per category for pass-through estimates were carbonates 4.0%; concentrates -2.8%; juices 3.8%; bottled waters -1.2%; sports and energy drinks 18%.

## Before and after design without a control group

### Alternative methods for impact effect estimation

#### Prais-Winsten regression

Alternative to the ARIMAX models for tax effect an alternative regression approach based on Prais-Winsten linear models to account to auto-correlated structure within the data was implemented. The regression model is specified as follow:

$P_{\mathrm{it}}=\theta Tax+\varphi pre+\lambda post+g\left( t \right)+\delta_{\mathrm{month}}+\varepsilon_{\mathrm{it}}.$ (1)

where $P_{it}$ is the weighted average price per product category *i* at the month *t*. “*Tax*” is the policy indicator variable and $g\left( t \right)$ is a polynomial to control for a general time trend, selected based on AIC. Seasonality is captured using a vector of monthly dummy variables represented by $\delta_{\mathrm{month}}$. In this model, the parameter $\theta$ captures the impact of the tax after it is introduced, $\gamma$ potential announcement effects, $\varphi$ the pre-taxation prices trends and $\lambda$ the post-taxation general trend capturing potential further medium-term effects of the policy. The idiosyncratic error is represented by $\varepsilon_{\mathrm{it}}$.

Results for $P_{\mathrm{it}}$ estimated based on equation (1) are presented in table 1. The results are highly consistent with our main model results (ARIMAX).

Table S9: Price effect (% change) of soft drink taxation implemented in Chile (AR1 models)

|  | Carbonates | | Liquid juice | | Concentrates | | Bottled water | | Energy and sport drinks | |
| --- | --- | --- | --- | --- | --- | --- | --- | --- | --- | --- |
| Implementation effect | **6,769**** | **9,447**** | **3,036**** | **2,779*** | 0,282 | 1,457 | **2,376*** | 1,448 | **1,606*** | 1,010 |
|  | (5,118 - 8,420) | (7,018 - 11,876) | (1,653 - 4,419) | (0,494 - 5,064) | (-2,780 - 3,345) | (-2,930 - 5,843) | (0,535 - 4,218) | (-1,441 - 4,337) | (0,022 - 3,190) | (-2,046 - 4,066) |
| Announcement effect |  | **2,256*** |  | -0,234 |  | 1,085 |  | -0,891 |  | -0,478 |
|  |  | (0,506 - 4,005) |  | (-1,877 - 1,409) |  | (-2,133 - 4,304) |  | (-3,005 - 1,222) |  | (-2,565 - 1,609) |
| Pre-tax trend (monthly) | 0,594 | 0,492 | 0,386 | 0,401 | **-1,100*** | **-1,197*** | 0,678 | **0,778*** | -0,527 | -0,532 |
|  | (-0,064 - 1,253) | (-0,136 - 1,119) | (-0,196 - 0,967) | (-0,190 - 0,992) | (-2,150 - -0,049) | (-2,287 - -0,108) | (-0,044 - 1,400) | (0,044 - 1,512) | (-1,397 - 0,342) | (-1,422 - 0,359) |
| Post-tax trend (monthly) | -0,410 | -0,164 | 0,170 | 0,143 | **1,297**** | **1,339**** | 0,301 | 0,215 | -0,677 | -0,763 |
|  | (-0,928 - 0,108) | (-0,685 - 0,357) | (-0,279 - 0,620) | (-0,348 - 0,635) | (0,324 - 2,270) | (0,348 - 2,330) | (-0,271 - 0,873) | (-0,395 - 0,824) | (-1,439 - 0,086) | (-1,652 - 0,126) |

## Before-after with control group design

### Interrupted Time-Series Analysis with control group

As an alternative sensibility analysis of our results and to explore other external effects in market prices non-attributable to the soft drink taxation, we estimated the effects in prices and affordability comparing the trends of the taxed products with a control group using an interrupted-time series analysis (ITSA) approach(4). The control group is composed by milks (non-alcoholic beverage product unaffected by the policy) and products of regular household consumption with stable demand and theoretically unrelated to the taxation (i.e. non-substitution effects are expected): dish detergents, laundry detergents, napkins and toilet paper.

$${\begin{aligned} Y \end{aligned}}_{\mathrm{it}}=\beta_{0}+\beta_{1}{Group}_{i}+ \beta_{2}Time+ \beta_{3}Group*Time+ \beta_{4}Tax+ \beta_{5}Tax*Group{+ \beta}_{6}PostTax{+ \beta}_{7}PostTax*Group+X+\epsilon_{\mathrm{it}}$$

Where ${\begin{aligned} Y \end{aligned}}_{\mathrm{jkt}}$ is either the price or the affordability of the product *i* in the time *t,* $\beta_{0}$ is the intercept, $\beta_{1}$ represent baseline differences between the treated and control group, $\beta_{2}$ captures the general time trend in prices, $\beta_{3}$ the interaction between time-trend and group capturing the differential trend of the taxed group, $\beta_{4}$ the general effect of the tax implementation in the outcome, $\beta_{5}$ captures the effect of the tax in the treated group, $\beta_{5}$ represent the effect of the taxation in the outcome trends in the post-tax period and $\beta_{7}$ is the post-tax effect in outcome trends in the taxed group. X is a vector of control covariables including wages index, unemployment, seasonal and annual dummy variables and a time polynomial until the fifth grade for a flexible adjustment of general time trends. $\epsilon_{\mathrm{it}}$ represent the error term. At least four different model specifications are tested to check the robustness of the results: fixed-effect model by product category (FE); mixed-effect model with random intercept by product category (ME); Prais-Winsten regression (AR1); and Prais-Winsten regression with correlated panels corrected standard errors (PSAR1).

Model results estimates are presented graphically in the following figures. Point estimates are presented in diamonds and 95% confidence intervals in solid lines. Results are consistent with the estimates from our main analysis (ARIMAX model).

Figure S10: Price effect (% change) on carbonates compared to controls after the taxation implemented in Chile

Point estimates are presented in diamonds and 95% confidence intervals in solid lines. Implementation effect capture the effect (% change) in the beverage category immediately after the tax policy was implemented.

Control group: milks, dish detergents, laundry detergents, napkins and toilet paper.

Models: Fixed-effect model by product category (FE); mixed-effect model with random intercept by product category (ME); Prais-Winsten regression (AR1); and Prais-Winsten regression with correlated panels corrected standard errors (PSAR1).

Figure S11: Affordability effect (% change) on carbonates compared to controls after the taxation implemented in Chile

Point estimates are presented in diamonds and 95% confidence intervals in solid lines. Implementation effect capture the effect (% change) in the beverage category immediately after the tax policy was implemented.

Control group: milks, dish detergents, laundry detergents, napkins and toilet paper.

Models: Fixed-effect model by product category (FE); mixed-effect model with random intercept by product category (ME); Prais-Winsten regression (AR1); and Prais-Winsten regression with correlated panels corrected standard errors (PSAR1).

Figure S12: Price effect (% change) on juice compared to controls after the taxation implemented in Chile

Point estimates are presented in diamonds and 95% confidence intervals in solid lines. Implementation effect capture the effect (% change) in the beverage category immediately after the tax policy was implemented.

Control group: milks, dish detergents, laundry detergents, napkins and toilet paper.

Models: Fixed-effect model by product category (FE); mixed-effect model with random intercept by product category (ME); Prais-Winsten regression (AR1); and Prais-Winsten regression with correlated panels corrected standard errors (PSAR1).

Figure S13: Affordability effect (% change) on juice compared to controls after the taxation implemented in Chile

Point estimates are presented in diamonds and 95% confidence intervals in solid lines. Implementation effect capture the effect (% change) in the beverage category immediately after the tax policy was implemented.

Control group: milks, dish detergents, laundry detergents, napkins and toilet paper.

Models: Fixed-effect model by product category (FE); mixed-effect model with random intercept by product category (ME); Prais-Winsten regression (AR1); and Prais-Winsten regression with correlated panels corrected standard errors (PSAR1).

Figure S14: Price effect (% change) on concentrates compared to controls after the taxation implemented in Chile

Point estimates are presented in diamonds and 95% confidence intervals in solid lines. Implementation effect capture the effect (% change) in the beverage category immediately after the tax policy was implemented.

Control group: milks, dish detergents, laundry detergents, napkins and toilet paper.

Models: Fixed-effect model by product category (FE); mixed-effect model with random intercept by product category (ME); Prais-Winsten regression (AR1); and Prais-Winsten regression with correlated panels corrected standard errors (PSAR1).

Figure S15: Affordability effect (% change) on concentrates compared to controls after the taxation implemented in Chile

Point estimates are presented in diamonds and 95% confidence intervals in solid lines. Implementation effect capture the effect (% change) in the beverage category immediately after the tax policy was implemented.

Control group: milks, dish detergents, laundry detergents, napkins and toilet paper.

Models: Fixed-effect model by product category (FE); mixed-effect model with random intercept by product category (ME); Prais-Winsten regression (AR1); and Prais-Winsten regression with correlated panels corrected standard errors (PSAR1).

Figure S16: Price effect (% change) on water compared to controls after the taxation implemented in Chile

Point estimates are presented in diamonds and 95% confidence intervals in solid lines. Implementation effect capture the effect (% change) in the beverage category immediately after the tax policy was implemented.

Control group: milks, dish detergents, laundry detergents, napkins and toilet paper.

Models: Fixed-effect model by product category (FE); mixed-effect model with random intercept by product category (ME); Prais-Winsten regression (AR1); and Prais-Winsten regression with correlated panels corrected standard errors (PSAR1).

Figure S16: Affordability effect (% change) on water compared to controls after the taxation implemented in Chile

Point estimates are presented in diamonds and 95% confidence intervals in solid lines. Implementation effect capture the effect (% change) in the beverage category immediately after the tax policy was implemented.

Control group: milks, dish detergents, laundry detergents, napkins and toilet paper.

Models: Fixed-effect model by product category (FE); mixed-effect model with random intercept by product category (ME); Prais-Winsten regression (AR1); and Prais-Winsten regression with correlated panels corrected standard errors (PSAR1).

# References

1. Chen C, Liu L-M. Joint Estimation of Model Parameters and Outlier Effects in Time Series. Journal of the American Statistical Association. marzo de 1993;88(421):284.

2. López-de-Lacalle J. tsoutliers: Detection of Outliers in Time Series. 2017.

3. Box GEP, Jenkins GM, Reinsel GC. Time Series Analysis. Fourth Edi. Hoboken, NJ: John Wiley & Sons, Inc.; 2008.

4. Linden A, Arbor A. Conducting interrupted time-series analysis for single-and multiple-group comparisons. The Stata Journal. 2015;15(2):480–500.
